# Aggregated responses of human mobility to severe winter storms: An empirical study

**DOI:** 10.1371/journal.pone.0188734

**Published:** 2017-12-07

**Authors:** Yan Wang, Qi Wang, John E. Taylor

**Affiliations:** 1 Charles E. Via, Jr. Department of Civil and Environmental Engineering, Virginia Tech, Blacksburg, VA, United States of America; 2 Department of Civil and Environmental Engineering, Northeastern University, Boston, MA, United States of America; 3 School of Civil and Environmental Engineering, Georgia Institute of Technology, Atlanta, GA, United States of America; East China University of Science and Technology, CHINA

## Abstract

Increasing frequency of extreme winter storms has resulted in costly damages and a disruptive impact on the northeastern United States. It is important to understand human mobility patterns during such storms for disaster preparation and relief operations. We investigated the effects of severe winter storms on human mobility during a 2015 blizzard using 2.69 million Twitter geolocations. We found that displacements of different trip distances and radii of gyration of individuals’ mobility were perturbed significantly. We further explored the characteristics of perturbed mobility during the storm, and demonstrated that individuals’ recurrent mobility does not have a higher degree of similarity with their perturbed mobility, when comparing with its similarity to non-perturbed mobility. These empirical findings on human mobility impacted by severe winter storms have potential long-term implications on emergency response planning and the development of strategies to improve resilience in severe winter storms.

## Introduction

Recent developments in information technology have provided an unprecedented amount of crowdsourced spatial-temporal data to study human mobility [1–5]. Findings about daily patterned human movements have fundamentally changed our understanding of human mobility at varying spatial scales. However, human mobility patterns under perturbed states, such as in natural disasters, also require a deeper understanding in order to prepare for unfamiliar conditions in the future [6]. Scholars in the disaster research area have identified scaling laws and evaluated the predictability of human mobility during and after extreme events using mobility patterns from non-perturbed states. Lu, et al. [7] used approximately one year of mobile phone data from 1.9 million users, and found that population movements following the Haiti earthquake had a high level of predictability, and destinations were correlated with normal-day mobility patterns and social support structure. Similar results have been found in the research of Song, et al. [8] on human mobility following the Great East Japan Earthquake and Fukushima nuclear accident. A study by Wang and Taylor [5] showed that human mobility was significantly perturbed during Hurricane Sandy but also exhibited high levels of resilience. A more recent study on multiple types of natural disasters revealed a more universal pattern of human mobility, as well as the limitations of urban human mobility resilience, under the influence of multiple types of natural disasters [9]. This study also uncovered that resilience could be significantly impacted by more powerful disasters, which could force urban residents to adopt entirely different travel patterns from their norms. Other scholars have conducted longitudinal studies on the relationship between large-scale natural disasters and long-term population mobility. For example, Gray and Mueller [10] investigated the effects of flooding and crop failures on local population mobility and long-distance migration over 15 years in rural Bangladesh. Their results revealed that natural disasters had significant effects on long-term population mobility but mobility did not universally serve as a post-disaster coping strategy.

Unlike other acute disasters (e.g., earthquakes, hurricanes, and floods), severe winter storms may not force residents to evacuate from their homes to safer places on a large scale, which may result in different perturbation patterns. Yet, relatively few studies have examined the relationship between winter hazards and human mobility in great detail. Over the past century, severe winter storms continue to occur with greater frequency in the eastern two-thirds of the contiguous United States [11]. The increased damages from these storms has resulted in costly and disruptive effects on people’s daily lives. Large accumulations of snowfall and ice can incur catastrophic effects on infrastructure systems [12], specifically, electrical system emergencies and disturbances, and transportation delays and closures [13], and further lead to communications breakdowns and public health issues. Studies in the transportation research area have examined impacts of snowstorms on traffic based on limited traffic modes at small scales. For example, snowstorms have been found to impact different dimensions of traffic, e.g. traffic demand, traffic safety, traffic operations and flow [14]. The impact varies by trip purposes [15, 16] and distances [14], types of vehicles, different areas [17], and time [18]. In terms of trip purposes, results from a survey [16] indicate that snow and stormy weather have the least impact on commuting (work, school) behavior: the work and location were the least frequently changed and the main change in commuting behavior was in timing of the trips; while for shopping trips and leisure tips, more than half of the responds chose to cancel the trip and even more changed route and location. However, it is still unclear what the quantitative relationship between peoples’ recurrent mobility (characterized by their frequent visited places) and perturbed mobility during the winter storm is. In addition to traffic, heavy snow has been shown to have a negative impact on foot travel frequencies [19]. However, these empirical studies based on a single transportation mode at a small scale do not represent the population well, and, in turn, are unlikely to reveal the overarching impact of large-scale storms. The severity of the damages from winter storms calls for innovative research, particularly a fundamental understanding of human behaviors and activity patterns with aggregated data to achieve more effective snowstorm preparation and to build more resilient cities.

Based on the findings of studies on human mobility in disasters and the impact of snowstorms on traffic, in this paper we tested four hypotheses: Hypothesis 1: Individuals’ displacements of different lengths can be significantly perturbed by a severe winter storm and the perturbation varies among different ranges of distances and distinct days of a week; Hypothesis 2: Human mobility patterns can be affected by a severe winter storm, as measured by radii of gyration and the shifting distance of center of mass; Hypothesis 3: Individuals’ frequently visited locations can better quantify their mobility patterns during a severe winter storm than non-perturbed patterns during normal days.

## Methods

We selected the January 2015 severe winter storm in the northeastern United States for the seasonality and high frequency of this type of damage in this area and its large-scale impact. This severe storm caused a snow emergency to be declared by the Federal Emergency Management Agency [20] during January 27 to 29 in six states, including New Hampshire, Massachusetts, Connecticut, Rhode Island, Maine and New Jersey. This winter hazard brought heavy snow to southern New England with blizzard conditions to much of Rhode Island and Massachusetts, beginning during the day on January 26 and lasting into the early morning hours of January 27. We narrowed the area within the spatial bounding box coordinates of Massachusetts due to the population distribution, and the statewide impact (latitude: 41.187 to 42.887, and longitude: -73.508 to -69.859). The storm duration was from January 26 to 28 in this area. Much of this affected area received two to three feet of snow and experienced severe winds with gusts over 70 mph [21]. The Category of Regional Snowfall Index (RSI, which estimates societal impacts of snowstorms within a region’s borders) [22] for this storm has an Index value of 6.158 [23], which indicates a major snowstorm. A statewide driving ban was issued and MBTA public transportation service was suspended, thousands of flights were canceled, and schools and activities observed weather-related cancellations for one or more days [21].

The raw data for this study is comprised of geotagged Tweets collected from a Twitter Streaming API [24]. We use geotagging as the only filter to collect real-time Tweets. Our data collection and usage comply with the Twitter Terms of Service. As 1.24% of Tweets are geotagged [25] and the streaming API can collect 1% of Tweets, the database of this study is representative in terms of geotagged Tweets. The Twitter geotags are based on GPS Standard Positioning Service which offers a worst-case pseudo-range accuracy of 7.8 meters with 95 percent confidence, and the positional accuracy are affected by weather and device factors [26]. The studied time period includes four pre-storm weeks, a during-storm week, and a post-storm week—from December 29, 2014 to February 8, 2015. The data volume for each day can be found in S1 Table. In total, 2,691,346 Tweets were collected over the 42 days and the average daily data volume was about 64,080 Tweets.

## Results

### Daily displacements

**Fig 1:**
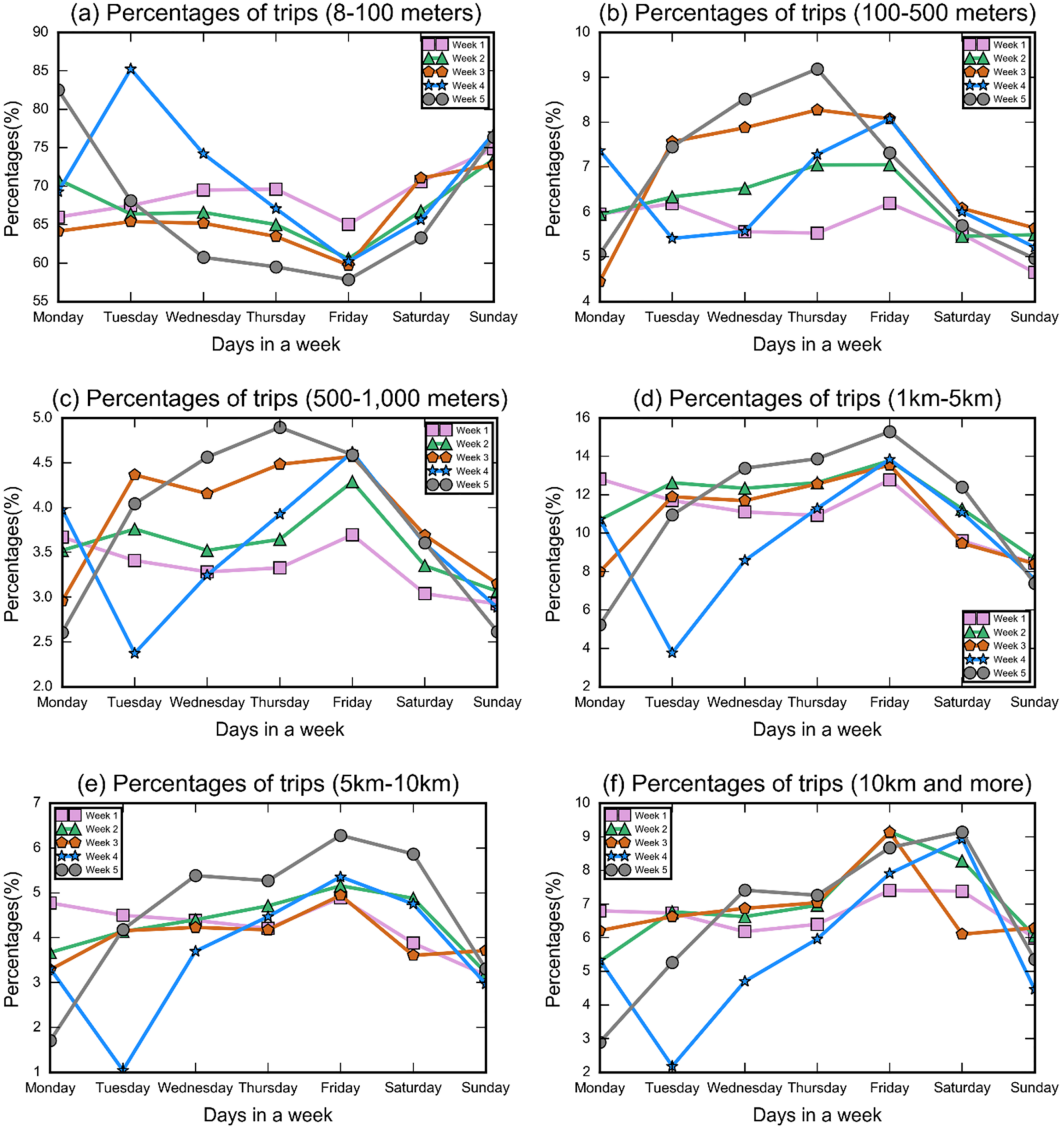
Impact of the severe winter storm on percentages of trips in different distance ranges. (a-f) Points in each line represent percentages of a trip on distinct days in a week. The star points in blue lines refer to percentages during the severe winter storm week (Week 4), when the Monday, Tuesday and Wednesday were the exact three-day duration of the storm. Weeks 1-3 are the weeks before the storm and Week 5 is the week after the storm. Counts of displacements in all ranges can be found in S2 Table.

To explore if severe winter storms can perturb people’s daily trajectories, displacements of each distinct user during thirty-five 24-hour periods over January 5 to February 8, 2015 (Eastern Time) were calculated and studied. Displacement in our studies is defined as the Haversine distance between two consecutive geolocations of an individual within a day. To avoid the inaccuracy of GPS services [26], we exclusively focused on displacements which are longer than eight meters. Six groups of distances were set, including 8-100 meters (*r*_1_), 100-500 meters (*r*_2_), 500-1,000 meters (*r*_3_), 1-5km (*r*_4_), 5-10km (*r*_5_), and 10km and more (*r*_6_). Data volume of displacements per day varied from 12,576 to 70,565 (see S2 Table). Percentages of the number of displacements within the six sets during pre-storm weeks and the storm week were then computed and plotted for comparison (see Fig 1).

The storm week (Week 4) exhibits a clear perturbation pattern for each group of distances. The four plots for normal weeks (Week 1, 2, 3, 5) exhibiting regularity are comparable within each group of distances. For example, during the normal weeks, the average percentages of short trips (*r*_1_) decreases from Monday (72.92%) to Friday (60.81%), and increases from Friday to Sunday (74.38%). However, during the storm week, heavy snow incurs more short trips and the percentage of short trips (*r*_1_) achieves the peak at 85.23% on Tuesday (the storm day) and decreases sharply to 60.20% on that Friday. It finally returns to a normal level (76.86%) on Sunday. In contrast, the long trips (*r*_6_) experience a decreasing trend to its lowest percentage on Tuesday (2.18%) compared with the increasing trend from Monday to Friday under normal circumstances. It rebounds to its highest value of 7.91% on Friday. Trips of other distance ranges also experienced substantial perturbation during the storm.

To quantitatively examine the observations from Fig 1, we adopted binary logistic regression to check if the severe winter storm statistically affected trips of different ranges. We set storm and non-storm statuses as binary explanatory variables (1 and 0 respectively), and percentages of trips of a distinct category as response variable. The coefficient and significance values can be found in S3 Table. We found that percentages of longer trips (i.e. *r*_4_, *r*_5_, and *r*_6_) and the shortest trip (*r*_1_) were statistically significantly influenced by the winter storm (p-value< 0.05). However, percentages of *r*_2_ and *r*_3_, although obvious decreased from the Monday to Tuesday during the storm week, were not statistically significantly changed by the snow storm over the three day period.

**Fig 2:**
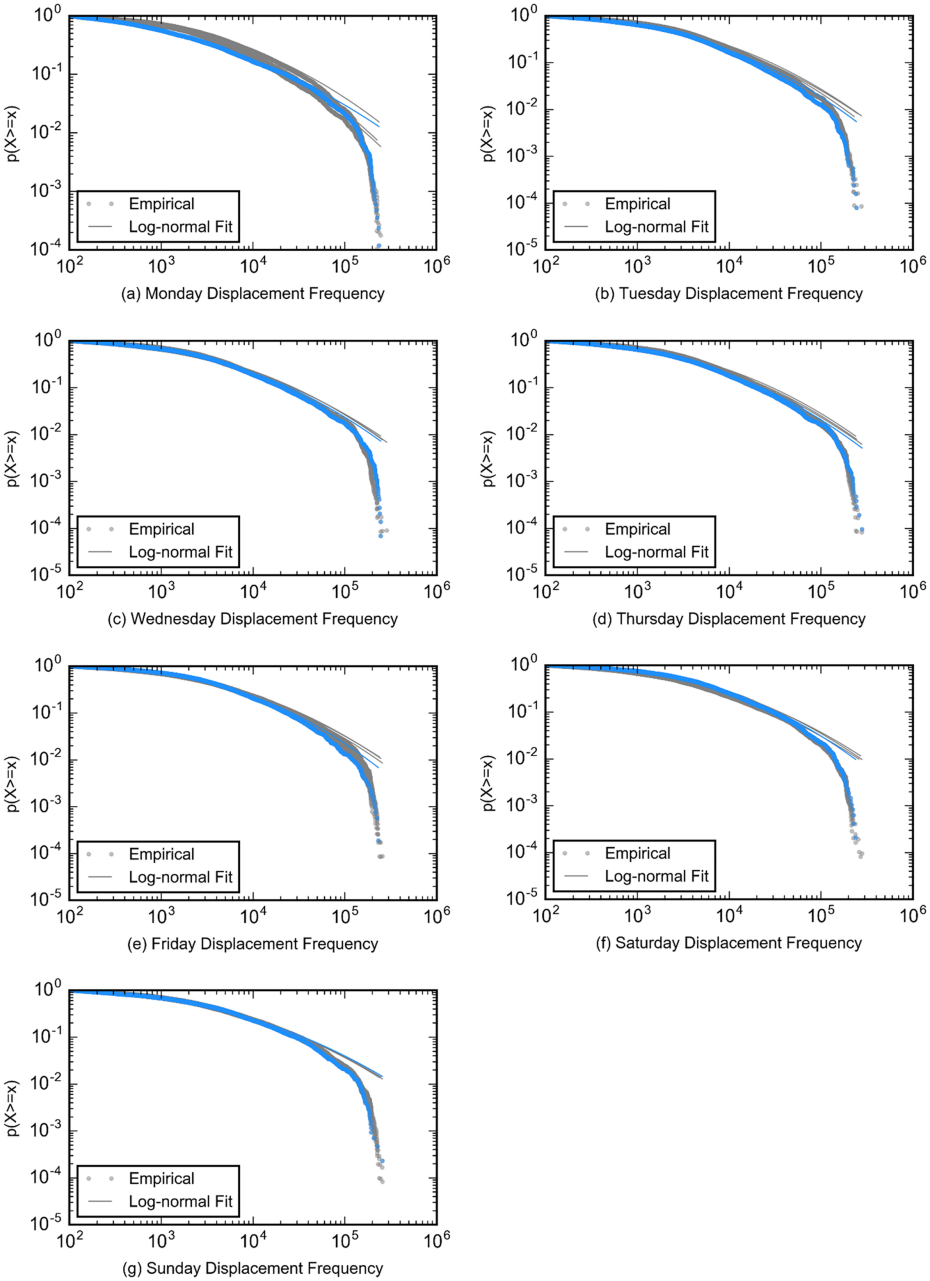
Complementary cumulative distribution function (CCDF) of displacements for empirical data and log-normal fitted data. (a-g) Graphs show the distributions of displacements from Mondays to Sundays during the studied weeks. The dashed lines in each graph represents the CCDF for empirical data, while the solid lines refer to the CCDF for log-normal fitted data. Blue lines represent the days in the storm-affected week, and other grey lines refer to days during normal weeks. The daily displacement distribution is well fitted with lognormal distribution.

To arrive at a detailed understanding of the daily displacements, we further fitted daily displacements from January 5 to February 8 into distributions including lognormal, exponential, and power law, using the Python package Powerlaw [27]. Lognormal distribution (Eq (1)) best characterized their distributions based on the loglikelihood ratio and the corresponding value. We plotted the complementary cumulative distribution of displacements for empirical data and log-normal fitted data according to different days in a week in Fig 2.

$$\begin{array}{c} P\left(x\right)∼\frac{1}{x\sigma \sqrt{2\pi }}e^{-\frac{{\left(Inx-\mu \right)}^{2}}{2\sigma^{2}}}\; \end{array}$$ (1)

The results of fitting and comparisons with other distributions are all included in S4 Table. For the fitted parameters, the values of mean (*μ*) in all fittings are in the range 7.452±0.560, while January 27 has the smallest mean value of 4.627. However, all the snowstorm days and the following days have relatively smaller mean values than normal days. The values of standard deviation (*σ*) in all fittings are in the range of 2.077±0.239 except for the most severe day of the storm, January 27, with the highest value of 3.232 and the following Monday (February 2) with value of 2.566, which indicates more differences among frequencies of different-length displacements. The displacements decay faster on that Monday perhaps because fewer people tended to travel longer distances due to the severe snow storm.

Both the results of the logistic regression and the scaling parameters from fitting log-normal distribution show that the severe winter storm has significantly impacted displacements, and the perturbation varies among distinct days of a week. Therefore, we find support for Hypothesis 1.

### Radii of gyration

Radii of gyration (*r*_*g*_), a measurement of object movement from physics, has been widely used to quantify the size of trajectory of individuals since the study of Gonzalez, et al. (2008) [2]. To achieve a more nuanced understanding of the perturbation of human mobility patterns, we computed the daily *r*_*g*_ of each distinct user from January 12 to February 8 to identify the change of daily radii of gyration over time. We adopted the formula in Eq (2) [9] to calculate the *r*_*g*_ of each distinct individual in the data set.

$$\begin{array}{c} r_{g}=\sqrt{\frac{1}{n}\sum_{t=1}^{n}[2r\times sin^{-1}(\sqrt{sin^{2}(\frac{\emptyset_{k}-\emptyset_{c}}{2})+cos\emptyset_{k}cos\emptyset_{c}sin^{2}(\frac{\varphi_{k}-\varphi_{c}}{2})})]} \end{array}$$ (2)

Where n is the total frequency of visited locations of one individual, *k* is each location visited by the individual during a certain period, *c* is the center of mass of trajectories, ∅ is the latitude, and *φ* is the longitude.

**Fig 3:**
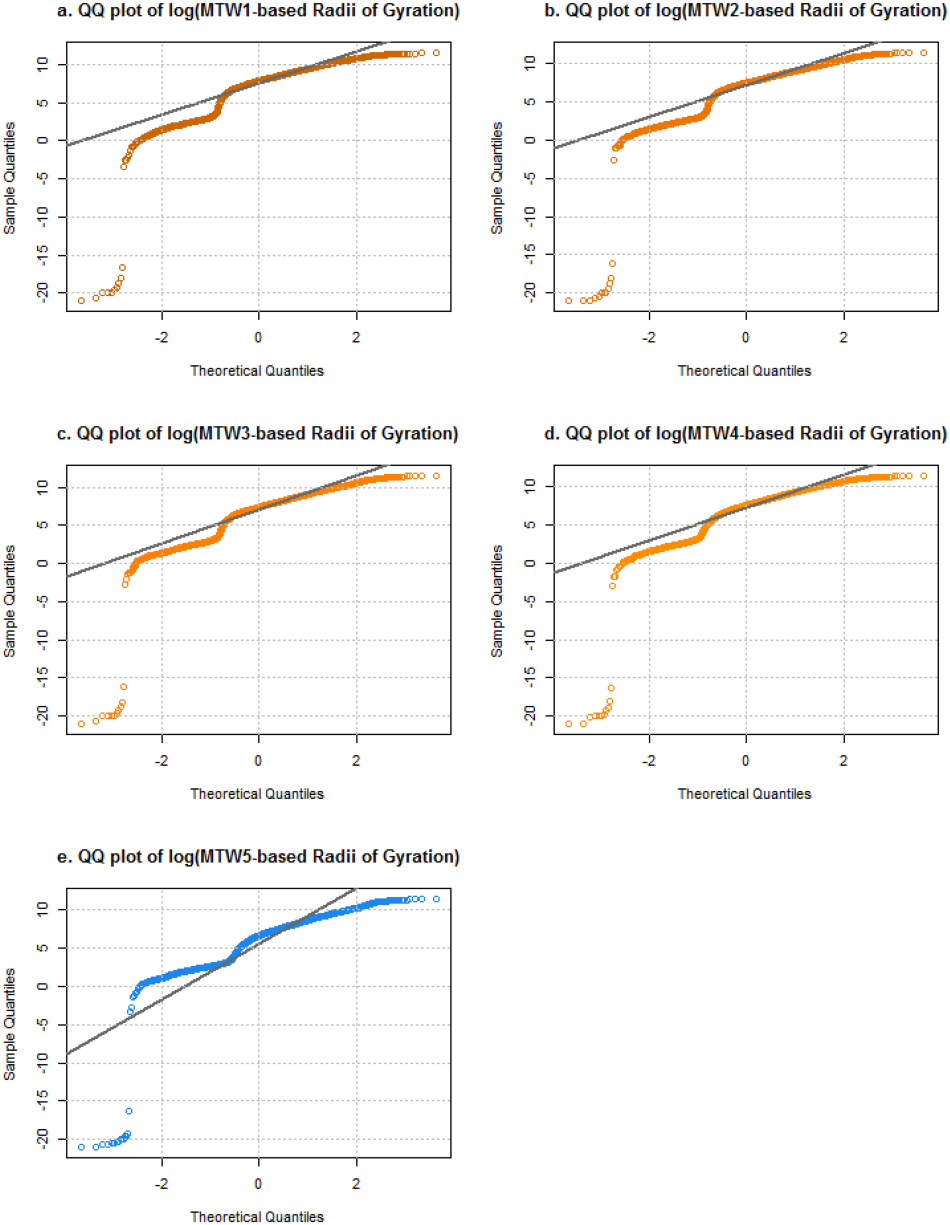
Comparison of empirical distributions of MTW-based between normal status and disaster status based on Quantile-Quantile plot. (a-e) These plots show a plot of the quantiles of the five data sets of MTW-based radii of gyration against the quantiles of the theoretical data set distributed as standard normal. The referenced straight lines pass through the first and third quartiles. MTW5 refers to the storm affected days. Its curved line and reference line show an obvious deviation from lines of other sets.

To minimize the variance among distinct days in a week and to better reflect the influence of the severe winter storm, we computed *r*_*g*_ based on a Monday, Tuesday and Wednesday (MTW-based *r*_*g*_) which were the three days (January 26 to 28) experiencing the most substantial effects of the winter storm. We also computed four sets of *r*_*g*_ for four MTWs in consecutive weeks from December 29, 2014 to January 25, 2015, before the storm. We filtered users to make sure each distinct user had at least two geolocations during each three-day period. This resulted in 3,743 distinct users with at least ten entries over the 15 days, and 95.18 average geotags per person. The total entries transmitted by the 3,743 users were 356,164, including 75,179 storm-day locations and 280,985 normal-day locations. We used Quantile-Quantile plots (also called Q-Q plots) to compare the distributions of MTW-based *r*_*g*_ among four pre-storm normal weeks and the storm week (Fig 3). The deviations between different pairs of MTW were quantified with a two-sample Kolmogorov-Smirnov test. The statistics and p-values can be found in S5 Table. The empirical distribution of MTW-based *r*_*g*_ during the snow storm week and during normal weeks has the largest value of deviation comparing with deviations between other pairs of distributions.

We further computed the daily *r*_*g*_ of each distinct user from January 12 to February 8 to identify the change of daily radii of gyration for the week before, during and after the winter hazard. We fitted the daily *r*_*g*_ to different distributions and found that *truncated power law* provides a better approximation of daily *r*_*g*_ than exponential and log-normal distributions. All the fits pass the Kolmogorov-Smirnov test for goodness of fit. The results can be found in S6 Table.

We used the *scaling parameter* (*α*) to evaluate the human mobility pattern as well as the perturbation duration. For the whole six weeks, *α* = 1.62 ± 0.17(mean±standard deviation), which is not far from the values of the scaling parameter identified in former studies [1, 2, 5]. For the four pre-storm weeks without any snow, *α* = 1.63 ± 0.05 (mean±standard deviation), which demonstrates a steady mobility pattern. The stable pattern also lasts until the beginning two days of the snow (January 26 and 27), however, *α* experiences its first peak at 1.78 on January 28, indicating more spread-out frequencies of all displacements. The values return back to a normal range in the next two days, but drop to the lowest points (1.00 and 1.41) in the weekends after the storm, which indicates a substantially changed mobility pattern. This may be caused by the increasing needs of individuals to take longer-distance trips to undertake activities that would have normally occurred in the weekdays when the heavy snow caused inconvenience for travel. Moreover, the relatively higher values of scaling parameters starting from the Thursday in the post-storm week may have been caused by the accumulation of snow on non-consecutive days (February 2, 3, 6, and 8) in some of the studied geographical area following the severe storm. Due to the severe winter storm, the snow depth examined in most areas included in this study exceeded ten inches [19]. The accumulated snow may have been the cause of the perturbed mobility patterns. Based on these results, we find support for Hypothesis 2.

### Shifting distance of center of mass

We computed the shifting distance of the center of mass (*Δd_CM_*) to quantify the change of mobility pattern. The average center of mass of distinct individuals under four normal statuses and one in the snowstorm period were calculated separately. The *Δd_CM_* was the shifting distance of the average center of mass from the normal state to the perturbed snowstorm state. Eq (3) was employed to calculate the shifting.

$$\begin{array}{c} \Delta d_{CM}=|{\overset{⃑}{r}}_{CM}^{S}-{\overset{⃑}{r}}_{CM}^{N}| \end{array}$$ (3)

Where ${\overset{⃑}{r}}_{CM}^{S}$ is the average center of mass of a movement trajectory during the storm days, and ${\overset{⃑}{r}}_{CM}^{N}$ is the average center of mass during the first four sets of Monday, Tuesday and Wednesday.

The *truncated power law* distribution was found to be the best distribution of *Δd_CM_* compared to lognormal and exponential distributions Eq (4). The parameters were obtained using the KS fit method. Fitting and comparison results can be found in Table 1.

$$\begin{array}{c} P\left(\Delta d_{CM}\right)\propto \Delta d_{CM}^{-1.3735}e^{-0.7396\Delta d_{CM}} \end{array}$$ (4)

**Table 1:** Truncated power law fitting and comparison results of *Δ*d_*CM*_.

*β* Value	*λ* Value	*κ* Value (m)	*KS*-test	Lognormal Comparison	p-value	Exponential Comparison	p-value
1.373	0.740	0.027	0.013	47.807	1.452e-30	1441.284	3.504e-55

### Relationship between perturbed mobility and recurrent mobility

The mobility patterns of individuals are dominated by their recurrent movement between a few primary locations [28, 29] and have a high predictability [7, 30, 31]. These most frequently visited locations include home, work, and school, along with several less active subsidiary locations [32, 33]. To examine if most frequented locations (MFLs) can quantify human mobility patterns under the severe winter storm as well, we compared the radii of gyration of MFLs ($r_{g}^{MFLs}$) with both $r_{g}^{n}$ (normal status) and $r_{g}^{s}$ (storm status) of each distinct individual. We defined the MFLs as the centroids of different clusters. Only users with at least two MFLs (two clusters) during normal days and at least two geolocations in a day under storm status were studied. MFLs of each distinct user were extracted from their four-week trajectories before the blizzard utilizing the DBSCAN algorithm. We set the required two input parameters for the clustering as follows: the maximum search radii was set as 20 meters, and the minimum number of points to form a cluster was set as two. The initial settings are based on the accuracy of the Twitter geotags, and the sensitivity analysis results on distance parameters of DBSCAN for Twitter data as identified in the study [26]. The MFLs of distinct individuals were then ranked according to their visitation frequencies, and MFLs with the same visitation frequencies have different but consecutive rankings.

**Fig 4:**
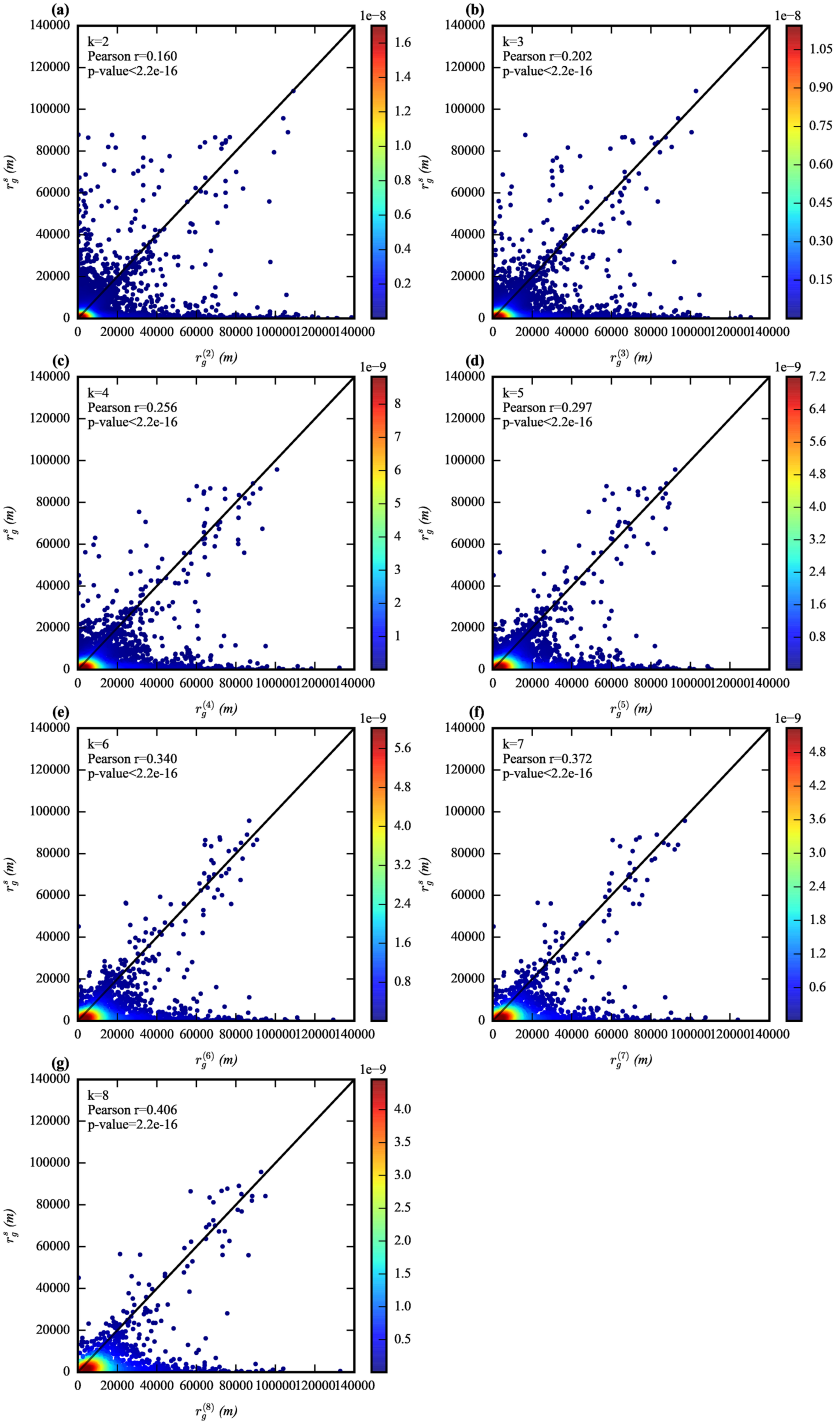
Comparisons between $r_{g}^{s}$ and $r_{g}^{\left(k\right)}$. (a-g) The scatter plots compared the correlation between $r_{g}^{\left(k\right)}$ and $r_{g}^{s}$ for *k* = 2, 3 … 7, 8 separately. Colors from blue to red represent the point density. With the increase of *k*, the radii of gyration of MFPs shows a stronger correlation with $r_{g}^{s}$ significantly based on the increasing value of Pearson *r*.

To quantify the human mobility pattern characterized by MFLs, we adopted the definition of the k-radii of gyration $r_{g}^{\left(k\right)}$ [33], which is the radii of gyration of *k*-th MFLs of an individual. The comparisons between $r_{g}^{\left(k\right)}$ and $r_{g}^{s}$ allows us to quantify the correlations between *k*-th MFLs and mobility pattern during the winter storm. We plotted the scatter graphs to observe the correlations with the point density which is colored from blue to red (Fig 4). We used Pearson correlation coefficient to quantify the strength of the correlation between $r_{g}^{\left(k\right)}$ and $r_{g}^{s}$. The value of Pearson correlation coefficient *r* and its corresponding *p* value are shown in Table 2. The *p* value is less than 0.01 for all comparisons, which indicates strong statistical significance. *r* is positive for each case. It ranges from 0.760 to 0.923 for the comparisons between $r_{g}^{n}$ and $r_{g}^{\left(k\right)}$, and 0.161 to 0.404 for the comparisons between $r_{g}^{s}$ and $r_{g}^{\left(k\right)}$ (*k* = 2,…,8). With the increasing value of *k*, the radii of gyration of MFLs presents a stronger correlation with both $r_{g}^{n}$ and $r_{g}^{s}$. However, by comparing the correlation coefficient *r* for both comparisons ($r_{g}^{n}$ and $r_{g}^{\left(k\right)}$, and $r_{g}^{s}$ and $r_{g}^{\left(k\right)}$) with the same *k* value, we found that the MFLs cannot characterize human mobility patterns during the severe winter storm better comparing with the higher similarity degree between recurrent mobility and mobility during the normal days. Therefore, Hypothesis 3 is rejected.

**Table 2:** Pearson’s correlation analysis between $r_{g}^{n}$ and $r_{g}^{\left(k\right)}$ and, between $r_{g}^{s}$ and $r_{g}^{\left(k\right)}$.

k	2	3	4	5	6	7	8
correlation coefficient ($r_{g}^{n}$, $r_{g}^{\left(k\right)}$)	0.7599454	0.8558787	0.8860109	0.8953719	0.9071972	0.9147848	0.9228992
*p*—*value*	<2.2e-16	<2.2e-16	<2.2e-16	<2.2e-16	<2.2e-16	<2.2e-16	<2.2e-16
correlation coefficient ($r_{g}^{s}$, $r_{g}^{\left(k\right)}$)	0.1604997	0.2023405	0.2562723	0.2973382	0.3395652	0.3723097	0.4060454
*p*—*value*	< 2.2e-16	2.2e-16	< 2.2e-16	< 2.2e-16	< 2.2e-16	< 2.2e-16	2.2e-16
*df*	11619	8025	5598	4004	2956	2237	1695

## Discussion

Previous research has found that natural disasters, e.g. hurricanes, floods and earthquakes, can cause significant impact on human mobility patterns [5–10]. We extend this research to severe winter storms showing that human mobility patterns are impacted in this different context. The results show that the severe winter storm caused significant perturbation on displacements in various ranges including short trips and long trips, which provide support to Hypothesis 1. The numbers of the shortest trips increased significantly while other longer trips decreased significantly. Similar findings have been found in single-mode transportation studies [14, 17–19]. This research builds upon these studies by examining large-scale empirical geo-temporal data, which may provide a more general perspective on human mobility. The high-accuracy geographical data also help to reveal specific impacts of a winter storm on displacements of different length. By investigating distribution of daily displacements over nearly one month, we found that daily displacements can be best approximated with the lognormal distribution under both normal and perturbed states. This result is consistent with the findings of Zhao, Musolesi [34] and Alessandretti, et al. [35], which focused on a normal, steady state mobility pattern. Parameters of the fitted lognormal distribution further help to examine the first hypothesis and the changed values over phases can signal the effect of the natural disaster.

The prediction of Hypothesis 2 that human mobility patterns would be affected is also supported: To minimize the variance between weekdays and weekends, we investigated distributions of radii of gyration on a Monday-Tuesday-Wednesday basis and distributions of daily radii of gyration. Distributions of both types of radii of gyration reflect the perturbation on mobility patterns caused by the severe storm. For Monday-Tuesday-Wednesday based radii of gyration, two-sample Kolmogorov-Smirnov tests uncovered the largest deviations between distribution during the storm week and distributions during the normal weeks. In terms of the daily radii of gyration, we found that this measurement can be best approximated by truncated power law during the severe winter storm and the truncated power law was found to be the dominant scaling law of mobility patterns in previous research [5, 9]. Scaling parameters of the fitted distributions help to detect the impact of the storm on the mobility pattern as well. To investigate the extent of the change of mobility pattern, we also measured the distance between center of mass of normal mobility and center of mass of perturbed mobility. The shifting distances fit a truncated power law distribution.

We further investigated the degree of similarity between recurrent mobility and mobility under normal and perturbed circumstances separately (Hypothesis 3). Although previous studies showed that individuals’ trajectories show a high degree of spatial regularity characterized by a few highly frequented locations [2, 28, 29, 31], the regularity does not remain during the severe winter storm. By comparing correlation between individuals’ recurrent mobility and perturbed mobility with correlation between recurrent mobility and normal mobility, we found that, contrary to Hypothesis 3, most frequented locations cannot better characterize individuals’ mobility pattern during the severe winter storm than during the normal circumstances. We also noticed that, for individuals with more recurrent locations, there is a higher correlation between recurrent mobility and perturbed mobility.

There are several limitations in this study deserving further research effort in the future. First, apart from the geotagged tweets used for this study, the self-reported locations in the text of tweets during disasters may also be included in future research data collection to achieve a broader sample. Only 1% of Twitter users geotag their tweets, but we still were able to evaluate 64,080 geotagged tweets per day, which is adequate for this analysis. Additionally, future analysis should examine the specific impact on human mobility of climate elements, such as snowfall and wind speed, and by expanding to multiple cases. This study narrowly examined the aggregated responses to the storm in a single case. Future research should examine how different geographical scales may influence the results. Regarding the relationship between recurrent mobility and normal and perturbed mobility, future research may examine and compare the specific semantic content of locations under different circumstances.

## Conclusion

This work contributes to a growing body of literature aimed at enhancing disaster resilience and risk management by understanding and predicting human mobility using crowdsourced data. The results evaluate overall and nuanced aspects of perturbation on mobility patterns. The quantitative approaches adopted in this study form a framework to examine the impact of natural disasters on human mobility patterns using geolocation data from social media. This framework can be used to assess both spatial and temporal aspects of urban mobility during disasters, to supplement evaluations of evacuation performance, and to track urban resilience to natural disasters. The findings of this study form an important step toward understanding human mobility during disasters. The investigated mobility patterns in this paper could be combined with detailed transportation and weather data and semantic content from geo-social networking platforms to inform governments and policymakers regarding specific disaster responses and relief strategies, for example, through improved resource allocation, emergency information diffusion, disease prevention, and evacuation in disasters.

## Supporting information

S1 Table Daily data volume of tweets in the studied area from December 29, 2014 to February 8, 2015. (DOC)

S2 Table Number of displacements in different ranges from January 5 to February 8, 2015. (DOC)

S3 Table Binary logistic regression results for examining the impact of the winter storm on percentages of different displacements. (DOC)

S4 Table Fitting parameters of lognormal distribution for daily displacements and comparison results with other distributions. (DOC)

S5 Table Kolmogorov-Smirnov test between the distributions of MTW-based radii of gyration during distinct weeks. (DOC)

S6 Table Fitting parameters of truncated power law and comparison results for daily radii of gyration. (DOC)
