# Supplementary material for: Aggregated responses of human mobility to severe winter storms: An empirical study
Source: PLoS One. 2017 Dec 7;12(12):e0188734. doi: 10.1371/journal.pone.0188734 (PMC5720675; doi:10.1371/journal.pone.0188734)
Supplement: S1 Table — (DOC) [file pone.0188734.s001.doc]

**S1 Table.** Daily data volume of Tweets in the studied area from December 29, 2014 to February 8, 2015.

| **Weeks** | **Days in weeks** | **Date (EST)** | **Number of**  **Tweets** | **Number of**  **Distinct Users** |
| --- | --- | --- | --- | --- |
| **Pre-storm week 1** | Monday | 12/29/2014 | 59564 | 12753 |
|  | Tuesday | 12/30/2014 | 60460 | 12755 |
|  | Wednesday | 12/31/2014 | 62887 | 14562 |
|  | Thursday | 1/1/2015 | 72706 | 15770 |
|  | Friday | 1/2/2015 | 61067 | 13177 |
|  | Saturday | 1/3/2015 | 72865 | 14540 |
|  | Sunday | 1/4/2015 | 75799 | 14541 |
| **Pre-storm week 2** | Monday | 1/5/2015 | 52208 | 12697 |
|  | Tuesday | 1/6/2015 | 64083 | 13235 |
|  | Wednesday | 1/7/2015 | 66628 | 13932 |
|  | Thursday | 1/8/2015 | 67121 | 14662 |
|  | Friday | 1/9/2015 | 59727 | 13628 |
|  | Saturday | 1/10/2015 | 74570 | 15519 |
|  | Sunday | 1/11/2015 | 73988 | 14617 |
| **Pre-storm week 3** | Monday | 1/12/2015 | 70435 | 13875 |
|  | Tuesday | 1/13/2015 | 62167 | 13564 |
|  | Wednesday | 1/14/2015 | 60550 | 13399 |
|  | Thursday | 1/15/2015 | 61696 | 14031 |
|  | Friday | 1/16/2015 | 55977 | 13739 |
|  | Saturday | 1/17/2015 | 59532 | 14305 |
|  | Sunday | 1/18/2015 | 83717 | 16645 |
| **Pre-storm week 4** | Monday | 1/19/2015 | 67110 | 14262 |
|  | Tuesday | 1/20/2015 | 66181 | 14428 |
|  | Wednesday | 1/21/2015 | 63096 | 14277 |
|  | Thursday | 1/22/2015 | 62077 | 29170 |
|  | Friday | 1/23/2015 | 56261 | 14518 |
|  | Saturday | 1/24/2015 | 65609 | 15488 |
|  | Sunday | 1/25/2015 | 71178 | 15266 |
| **Storm week** | Monday | 1/26/2015 | 78878 | 16495 |
|  | Tuesday | 1/27/2015 | 68706 | 16344 |
|  | Wednesday | 1/28/2015 | 42541 | 11440 |
|  | Thursday | 1/29/2015 | 53300 | 13375 |
|  | Friday | 1/30/2015 | 61432 | 14726 |
|  | Saturday | 1/31/2015 | 61652 | 14964 |
|  | Sunday | 2/1/2015 | 124227 | 21421 |
| **Post-storm week** | Monday | 2/2/2015 | 88891 | 16398 |
|  | Tuesday | 2/3/2015 | 72425 | 15031 |
|  | Wednesday | 2/4/2015 | 68466 | 15615 |
|  | Thursday | 2/5/2015 | 49624 | 13146 |
|  | Friday | 2/6/2015 | 26745 | 9653 |
|  | Saturday | 2/7/2015 | 28156 | 10017 |
|  | Sunday | 2/8/2015 | 37044 | 11230 |
