# Supplementary material for: Aggregated responses of human mobility to severe winter storms: An empirical study
Source: PLoS One. 2017 Dec 7;12(12):e0188734. doi: 10.1371/journal.pone.0188734 (PMC5720675; doi:10.1371/journal.pone.0188734)
Supplement: S2 Table — (DOC) [file pone.0188734.s002.doc]

# S2 Table. Number of displacements in different ranges from January 5 to February 8, 2015.

| **Weekday** | **Date** | **8-100m** | **100-500m** | **500-1,000m** | **1km-5km** | **5km-10km** | **>=10km** | **Sum** |
| --- | --- | --- | --- | --- | --- | --- | --- | --- |
| Monday | 1/5/2015 | 18453 | 1664 | 1026 | 3585 | 1335 | 1901 | 27964 |
| Tuesday | 1/6/2015 | 24393 | 2235 | 1232 | 4228 | 1626 | 2434 | 36148 |
| Wednesday | 1/7/2015 | 26144 | 2091 | 1235 | 4178 | 1650 | 2325 | 37623 |
| Thursday | 1/8/2015 | 25635 | 2035 | 1225 | 4022 | 1549 | 2356 | 36822 |
| Friday | 1/9/2015 | 21156 | 2014 | 1202 | 4153 | 1590 | 2409 | 32524 |
| Saturday | 1/10/2015 | 29462 | 2296 | 1268 | 4009 | 1619 | 3082 | 41736 |
| Sunday | 1/11/2015 | 30471 | 1892 | 1191 | 3431 | 1289 | 2438 | 40712 |
| Monday | 1/12/2015 | 27329 | 2291 | 1358 | 4123 | 1416 | 2042 | 38559 |
| Tuesday | 1/13/2015 | 22265 | 2124 | 1261 | 4234 | 1387 | 2271 | 33542 |
| Wednesday | 1/14/2015 | 21992 | 2155 | 1163 | 4073 | 1453 | 2188 | 33024 |
| Thursday | 1/15/2015 | 21491 | 2329 | 1205 | 4174 | 1557 | 2302 | 33058 |
| Friday | 1/16/2015 | 18053 | 2100 | 1278 | 4104 | 1536 | 2725 | 29796 |
| Saturday | 1/17/2015 | 21048 | 1720 | 1056 | 3549 | 1538 | 2611 | 31522 |
| Sunday | 1/18/2015 | 33270 | 2485 | 1388 | 3931 | 1457 | 2736 | 45267 |
| Monday | 1/19/2015 | 25938 | 1799 | 5623 | 3233 | 1330 | 2511 | 35872 |
| Tuesday | 1/20/2015 | 23259 | 2690 | 1552 | 4230 | 1478 | 2356 | 35565 |
| Wednesday | 1/21/2015 | 22121 | 2672 | 1410 | 3968 | 1435 | 2332 | 33938 |
| Thursday | 1/22/2015 | 20973 | 2733 | 1481 | 4151 | 1380 | 2326 | 33044 |
| Friday | 1/23/2015 | 17228 | 2329 | 1318 | 3906 | 1426 | 2633 | 28840 |
| Saturday | 1/24/2015 | 24026 | 2055 | 1248 | 3202 | 1219 | 2064 | 33814 |
| Sunday | 1/25/2015 | 27844 | 2157 | 1205 | 3216 | 1421 | 2408 | 38251 |
| Monday | 1/26/2015 | 27534 | 2927 | 1580 | 4260 | 1315 | 2120 | 39736 |
| Tuesday | 1/27/2015 | 35824 | 2273 | 998 | 1583 | 439 | 917 | 42034 |
| Wednesday | 1/28/2015 | 16096 | 1207 | 704 | 1859 | 802 | 1020 | 21688 |
| Thursday | 1/29/2015 | 18565 | 2014 | 1086 | 3123 | 1237 | 1650 | 27675 |
| Friday | 1/30/2015 | 19351 | 2595 | 1484 | 4451 | 1722 | 2542 | 32145 |
| Saturday | 1/31/2015 | 20941 | 1915 | 1147 | 3531 | 1516 | 2848 | 31898 |
| Sunday | 2/1/2015 | 54238 | 3678 | 2034 | 5369 | 2097 | 3149 | 70565 |
| Monday | 2/2/2015 | 39725 | 2434 | 1255 | 2514 | 822 | 1393 | 48143 |
| Tuesday | 2/3/2015 | 26976 | 2951 | 1601 | 4336 | 1656 | 2084 | 39604 |
| Wednesday | 2/4/2015 | 22490 | 3152 | 1689 | 4954 | 1993 | 2744 | 37022 |
| Thursday | 2/5/2015 | 15259 | 2355 | 1255 | 3558 | 1352 | 1862 | 25641 |
| Friday | 2/6/2015 | 7276 | 920 | 577 | 1922 | 790 | 1091 | 12576 |
| Saturday | 2/7/2015 | 8265 | 744 | 471 | 1619 | 766 | 1194 | 13059 |
| Sunday | 2/8/2015 | 13869 | 901 | 475 | 1341 | 601 | 973 | 18160 |
