# Supplementary material for: Aggregated responses of human mobility to severe winter storms: An empirical study
Source: PLoS One. 2017 Dec 7;12(12):e0188734. doi: 10.1371/journal.pone.0188734 (PMC5720675; doi:10.1371/journal.pone.0188734)
Supplement: S3 Table — (DOC) [file pone.0188734.s003.doc]

**S3 Table.** Binary logistic regression results for examining the impact of the winter storm on percentages of different displacements.

| Displacements | $r_{1}$ | $r_{2}$ | $r_{3}$ | $r_{4}$ | $r_{5}$ | $r_{6}$ |
| --- | --- | --- | --- | --- | --- | --- |
| Coefficients | 0.2095 | -0.281 | -1.4 | -0.4933 | -1.3461 | -1.1236 |
| $p-value$ | 0.0472 * | 0.626 | 0.23 | 0.0495 * | 0.0348 * | 0.0257 * |

*** *p*-value <0.05, ** p-value <0.01, *** p-value <0.001**
