# Supplementary material for: Aggregated responses of human mobility to severe winter storms: An empirical study
Source: PLoS One. 2017 Dec 7;12(12):e0188734. doi: 10.1371/journal.pone.0188734 (PMC5720675; doi:10.1371/journal.pone.0188734)
Supplement: S4 Table — (DOC) [file pone.0188734.s004.doc]

**S4 Table.** Fitting parameters of lognormal distribution for daily displacements and comparison results with other distributions

| **Date** | $\mu$ | $\sigma$ | **x-min** | **KS-test** | **Exponential**  **Comparison** | $p-value$ | **Power law**  **Comparison** | $p-value$ |
| --- | --- | --- | --- | --- | --- | --- | --- | --- |
| 1/5/2015 | 7.714074 | 1.8839467 | 100 | 0.0268917 | 8424.3149 | 7.48E-110 | 2718.9939 | 0 |
| 1/6/2015 | 7.633188 | 1.9864321 | 100 | 0.0271221 | 9451.5004 | 7.43E-123 | 3182.6568 | 0 |
| 1/7/2015 | 7.6415128 | 1.9697795 | 100 | 0.0268014 | 9905.9043 | 7.80E-123 | 3160.3226 | 0 |
| 1/8/2015 | 7.7009732 | 1.969914 | 100 | 0.026715 | 9437.3211 | 1.19E-115 | 3095.3377 | 0 |
| 1/9/2015 | 7.697949 | 1.977473 | 100 | 0.02578 | 9035.4759 | 2.95E-125 | 3204.3304 | 0 |
| 1/10/2015 | 7.7276292 | 2.0823367 | 100 | 0.021631 | 10225.788 | 5.40E-164 | 3273.7879 | 0 |
| 1/11/2015 | 7.6507344 | 2.1156943 | 100 | 0.0170946 | 10020.655 | 3.00E-149 | 2627.4689 | 0 |
| 1/12/2015 | 7.4736826 | 1.939222 | 100 | 0.0193051 | 11042.463 | 1.36E-117 | 2807.4205 | 0 |
| 1/13/2015 | 7.6693953 | 1.8912733 | 100 | 0.0217776 | 9858.674 | 4.73E-123 | 3099.5574 | 0 |
| 1/14/2015 | 7.5939045 | 1.9969652 | 100 | 0.0280066 | 10148.909 | 9.85E-123 | 2854.0504 | 0 |
| 1/15/2015 | 7.5654991 | 1.986476 | 100 | 0.0220747 | 10663.181 | 3.00E-123 | 2998.6752 | 0 |
| 1/16/2015 | 7.7598972 | 2.0050847 | 100 | 0.0198165 | 11420.769 | 1.11E-149 | 3222.2293 | 0 |
| 1/17/2015 | 7.8926021 | 1.974368 | 100 | 0.0185425 | 8150.6348 | 2.09E-135 | 3187.9657 | 0 |
| 1/18/2015 | 7.5029216 | 2.1834179 | 100 | 0.0159464 | 13965.906 | 6.79E-178 | 2732.1952 | 0 |
| 1/19/2015 | 7.7026855 | 2.1325026 | 100 | 0.0177264 | 10604.086 | 6.92E-155 | 2513.1236 | 0 |
| 1/20/2015 | 7.3805641 | 2.074052 | 100 | 0.0181226 | 13356.736 | 2.80E-138 | 2794.5249 | 0 |
| 1/21/2015 | 7.3624088 | 2.1126258 | 100 | 0.0181609 | 13754.039 | 8.81E-145 | 2564.8933 | 0 |
| 1/22/2015 | 7.3774537 | 2.039341 | 100 | 0.0135814 | 14028.924 | 1.30E-139 | 2699.9003 | 0 |
| 1/23/2015 | 7.5668989 | 2.0901793 | 100 | 0.0153008 | 11862.467 | 6.00E-155 | 2842.0584 | 0 |
| 1/24/2015 | 7.4675597 | 2.1152317 | 100 | 0.016239 | 12047.723 | 1.72E-136 | 2212.4427 | 0 |
| 1/25/2015 | 7.5634346 | 2.1752502 | 100 | 0.0160784 | 12723.146 | 6.61E-163 | 2386.8762 | 0 |
| 1/26/2015 | 7.1689987 | 2.1092831 | 100 | 0.0172147 | 14074.603 | 9.39E-128 | 2484.805 | 0 |
| 1/27/2015 | 4.6271196 | 3.2318684 | 100 | 0.0176299 | 24291.392 | 7.57E-104 | 364.05651 | 1.16E-78 |
| 1/28/2015 | 7.4620614 | 2.0056545 | 100 | 0.0254057 | 6919.1801 | 4.10E-66 | 1269.0605 | 1.56E-191 |
| 1/29/2015 | 7.4374064 | 1.9638626 | 100 | 0.0219782 | 9232.4775 | 1.90E-90 | 2166.5594 | 0 |
| 1/30/2015 | 7.5464953 | 1.9917914 | 100 | 0.0191083 | 14055.495 | 3.13E-154 | 3164.3035 | 0 |
| 1/31/2015 | 7.8242904 | 2.1045986 | 100 | 0.0184183 | 10747.275 | 1.82E-158 | 2996.9974 | 0 |
| 2/1/2015 | 7.3307505 | 2.0970269 | 100 | 0.0180372 | 17857.974 | 4.45E-191 | 3593.8176 | 0 |
| 2/2/2015 | 6.3714917 | 2.5656001 | 100 | 0.0141697 | 18711.605 | 1.09E-127 | 1048.058 | 1.04E-211 |
| 2/3/2015 | 7.3102283 | 1.9786274 | 100 | 0.0207702 | 13606.764 | 4.93E-128 | 2801.5288 | 0 |
| 2/4/2015 | 7.4882972 | 1.992182 | 100 | 0.0211203 | 16076.138 | 4.10E-155 | 3480.1326 | 0 |
| 2/5/2015 | 7.3837635 | 1.9893356 | 100 | 0.0177213 | 11942.932 | 7.69E-110 | 2367.748 | 0 |
| 2/6/2015 | 7.7729563 | 1.8456478 | 100 | 0.0223493 | 3757.4646 | 2.46E-53 | 1619.6998 | 2.09E-264 |
| 2/7/2015 | 7.9685543 | 1.8790777 | 100 | 0.0200402 | 3540.4139 | 2.84E-58 | 1574.2667 | 1.28E-273 |
| 2/8/2015 | 7.4656352 | 2.2436281 | 100 | 0.0241779 | 4709.3316 | 3.02E-63 | 951.9652 | 8.26E-168 |

Note: μ: location parameter; σ: shape parameter.
