# Supplementary material for: Aggregated responses of human mobility to severe winter storms: An empirical study
Source: PLoS One. 2017 Dec 7;12(12):e0188734. doi: 10.1371/journal.pone.0188734 (PMC5720675; doi:10.1371/journal.pone.0188734)
Supplement: S5 Table — (DOC) [file pone.0188734.s005.doc]

**S5 Table.** Kolmogorov-Smirnov test between the distributions of MTW-based $r_{g}$of distinct weeks.

| **D** | **MTW1** | **MTW2** | **MTW3** | **MTW4** | **MTW5** |
| --- | --- | --- | --- | --- | --- |
| **MTW1** | -- | 0.09861*** | 0.11277*** | 0.061999*** | 0.23704*** |
| **MTW2** | 0.09861*** | -- | 0.035275* | 0.048103*** | 0.17397*** |
| **MTW3** | 0.11277*** | 0.035275*** | -- | 0.055318*** | 0.15473*** |
| **MTW4** | 0.061999*** | 0.048103*** | 0.055318*** | -- | 0.18573*** |
| **MTW5** | 0.23704*** | 0.17397*** | 0.15473*** | 0.18573*** | -- |

*** *p*-value <0.05, ** p-value <0.01, *** p-value <0.001**
