# Supplementary material for: Aggregated responses of human mobility to severe winter storms: An empirical study
Source: PLoS One. 2017 Dec 7;12(12):e0188734. doi: 10.1371/journal.pone.0188734 (PMC5720675; doi:10.1371/journal.pone.0188734)
Supplement: S6 Table — (DOC) [file pone.0188734.s006.doc]

**S6 Table**. Fitting parameters of truncated power law and comparison results for daily radii of gyration.

| **Date** | $\alpha$ | **λ Value** | $x_{\min}$ | **KS**  **test** | **Exponential comparison** | $p-value$ | **Lognormal comparison** | $p-value$ |
| --- | --- | --- | --- | --- | --- | --- | --- | --- |
| 12/29/2014 | 1.677792 | 0.027186 | 1 | 0.003789 | 8792.695 | 7.27E-103 | 241.4795 | 1.24E-53 |
| 12/30/2014 | 1.64596 | 0.029858 | 1 | 0.004748 | 8698.552 | 2.51E-117 | 218.7952 | 1.37E-47 |
| 12/31/2014 | 1.682389 | 0.032819 | 1 | 0.0044 | 9245.274 | 4.20E-127 | 312.0221 | 2.27E-90 |
| 1/1/2015 | 1.617259 | 0.03285 | 1 | 0.004269 | 9711.858 | 9.60E-158 | 312.6552 | 1.65E-93 |
| 1/2/2015 | 1.639829 | 0.033407 | 1 | 0.005323 | 8903.266 | 5.35E-98 | 216.74 | 7.74E-26 |
| 1/3/2015 | 1.654193 | 0.024691 | 1 | 0.00334 | 10580 | 1.31E-115 | 274.0301 | 1.92E-62 |
| 1/4/2015 | 1.564084 | 0.029839 | 1 | 0.004329 | 9560.395 | 3.93E-141 | 297.7152 | 1.44E-70 |
| 1/5/2015 | 1.640834 | 0.034999 | 1 | 0.004595 | 7927.065 | 1.31E-121 | 210.2639 | 3.01E-43 |
| 1/6/2015 | 1.547737 | 0.044362 | 1 | 0.006098 | 7403.094 | 3.30E-121 | 282.7266 | 2.03E-42 |
| 1/7/2015 | 1.533663 | 0.039952 | 1 | 0.005688 | 8020.675 | 4.81E-128 | 291.7857 | 9.82E-46 |
| 1/8/2015 | 1.674923 | 0.029134 | 1 | 0.004265 | 9898.8 | 1.81E-106 | 295.7366 | 3.61E-56 |
| 1/9/2015 | 1.662929 | 0.033653 | 1 | 0.003962 | 8575.292 | 2.13E-103 | 287.6484 | 9.36E-55 |
| 1/10/2015 | 1.536642 | 0.039045 | 1 | 0.005073 | 9075.244 | 5.77E-80 | 316.9192 | 7.31E-17 |
| 1/11/2015 | 1.616772 | 0.027208 | 1 | 0.003814 | 10147.2 | 1.92E-91 | 272.8236 | 7.14E-29 |
| 1/12/2015 | 1.550665 | 0.035764 | 1 | 0.005885 | 9183.866 | 1.41E-97 | 263.7783 | 1.51E-23 |
| 1/13/2015 | 1.603237 | 0.037261 | 1 | 0.005898 | 8383.99 | 1.82E-111 | 286.6831 | 7.11E-46 |
| 1/14/2015 | 1.619396 | 0.036396 | 1 | 0.0048 | 8345.182 | 5.44E-110 | 258.482 | 5.15E-41 |
| 1/15/2015 | 1.624642 | 0.039199 | 1 | 0.005466 | 8603.699 | 1.73E-111 | 284.5446 | 7.81E-42 |
| 1/16/2015 | 1.672148 | 0.039928 | 1 | 0.005063 | 7998.887 | 1.88E-130 | 292.919 | 2.47E-75 |
| 1/17/2015 | 1.737222 | 0.032074 | 1 | 0.004111 | 9578.312 | 3.48E-109 | 267.3032 | 1.65E-56 |
| 1/18/2015 | 1.601419 | 0.029386 | 1 | 0.004105 | 11182.97 | 4.78E-141 | 342.8838 | 1.67E-69 |
| 1/19/2015 | 1.678987 | 0.026487 | 1 | 0.00354 | 10054.41 | 3.32E-127 | 232.9802 | 5.02E-61 |
| 1/20/2015 | 1.570153 | 0.04039 | 1 | 0.006769 | 8553.414 | 5.45E-122 | 303.9375 | 3.11E-47 |
| 1/21/2015 | 1.609329 | 0.038337 | 1 | 0.004666 | 8376.04 | 3.79E-130 | 315.1208 | 3.41E-71 |
| 1/22/2015 | 1.632637 | 0.038934 | 1 | 0.004505 | 8426.792 | 5.69E-134 | 319.238 | 1.80E-79 |
| 1/23/2015 | 1.715587 | 0.039831 | 1 | 0.004316 | 8452.071 | 2.14E-111 | 310.4018 | 7.42E-61 |
| 1/24/2015 | 1.645239 | 0.03889 | 1 | 0.004671 | 9125.734 | 2.41E-144 | 332.9112 | 4.80E-84 |
| 1/25/2015 | 1.658536 | 0.030503 | 1 | 0.005517 | 10392.84 | 5.33E-137 | 318.3105 | 9.82E-82 |
| 1/26/2015 | 1.624413 | 0.037256 | 1 | 0.005383 | 20542.45 | 3.75E-270 | 639.595 | 9.08E-99 |
| 1/27/2015 | 1.679003 | 0.03759 | 1 | 0.005582 | 10202.91 | 1.25E-137 | 347.8261 | 9.74E-73 |
| 1/28/2015 | 1.784641 | 0.037292 | 1 | 0.004356 | 6975.379 | 1.15E-85 | 221.6139 | 4.84E-43 |
| 1/29/2015 | 1.73854 | 0.03326 | 1 | 0.003584 | 8190.559 | 3.43E-94 | 275.3367 | 1.50E-53 |
| 1/30/2015 | 1.710285 | 0.035059 | 1 | 0.004757 | 9631.311 | 6.37E-68 | 263.7794 | 3.12E-17 |
| 1/31/2015 | 1.000015 | 0.016048 | 1 | 0.190696 | 1537.415 | 3.40E-08 | 1456.877 | 8.56E-18 |
| 2/1/2015 | 1.404604 | 0.035657 | 1 | 0.004393 | 11856.34 | 2.08E-206 | 533.3147 | 5.67E-88 |
| 2/2/2015 | 1.577818 | 0.027653 | 1 | 0.005359 | 11876.23535 | 1.30E-134 | 313.5181823 | 8.83E-50 |
| 2/3/2015 | 1.640446 | 0.029665 | 1 | 0.004402 | 10427.03535 | 6.57E-73 | 292.4733557 | 1.26E-20 |
| 2/4/2015 | 1.609433 | 0.040030 | 1 | 0.005186 | 9206.847724 | 9.72E-57 | 327.7400074 | 9.92E-13 |
| 2/5/2015 | 1.791092 | 0.032968 | 1 | 0.003510 | 8064.003338 | 7.14E-60 | 262.8304351 | 8.54E-27 |
| 2/6/2015 | 1.902266 | 0.057567 | 1 | 0.003153 | 4349.457135 | 7.46E-77 | 240.5248181 | 6.20E-69 |
| 2/7/2015 | 1.942033 | 0.050031 | 1 | 0.003159 | 4835.736536 | 6.50E-86 | 205.2845631 | 2.75E-61 |
| 2/8/2015 | 1.000012 | 0.322756 | 1 | 0.074552 | 3050.553846 | 1.71E-268 | -2107.913365 | 2.74E-64 |
